# Friends or foes? Novel antimicrobials tackling MDR/XDR Gram-negative bacteria: a systematic review

**DOI:** 10.3389/fmicb.2024.1385475

**Published:** 2024-05-10

**Authors:** Mihai Octavian Dan, Daniela Tǎlǎpan

**Affiliations:** ^1^Department of Microbiology, Carol Davila University of Medicine and Pharmacy, Bucharest, Romania; ^2^Microbiology Laboratory, “Matei Bals” National Institute of Infectious Diseases, Bucharest, Romania

**Keywords:** salvage therapy, antibiotic resistance, ceftazidime/avibactam, imipenem/relebactam, ceftolozane/tazobactam, cefiderocol, aztreonam/avibactam, sulbactam/durlobactam

## Abstract

Gram-negative bacteria have been one of the most studied classes in the field of microbiology, especially in the context of globally alarming antimicrobial resistance levels to these pathogens over the course of the past decades. With high numbers of these microorganisms being described as multidrug-resistant (MDR), or even extended-drug-resistant (XDR) bacteria, specialists in the field have been struggling to keep up with higher prevalence of difficult-to-treat infections caused by such superbugs. The FDA approval of novel antimicrobials, such as cefiderocol (FDC), ceftolozane/tazobactam (C/T), ceftazidime/avibactam (CZA), imipenem/relebactam (IMR), sulbactam/durlobactam (SUL-DUR) and phase 3 clinical trials’ results of aztreonam/avibactam (ATM-AVI) has proven that, while all these substances provide encouraging efficacy rates, antibiotic resistance keeps up with the pace of drug development. Microorganisms have developed more extensive mechanisms of resistance in order to target the threat posed by these novel antimicrobials, thus equiring researchers to be on a constant lookout for other potential drug candidates and molecule development. However, these strategies require a proper understanding of bacterial resistance mechanisms to gain a comprehensive outlook on the issue. The present review aims to highlight these six antibiotic agents, which have brought hope to clinicians during the past decade, discussing general properties of these substances, as well as mechanisms and patterns of resistance, while also providing a short overview on further directions in the field.

**Systematic review registration:**

https://www.crd.york.ac.uk/prospero/#searchadvanced, Identifier CRD42024505832.

## 1 Introduction

Emergent antimicrobial resistance stands as a global public health issue that burdens clinicians, patients, and healthcare systems overall. Since the market approval of penicillin more than 80 years ago, the field of antibiotics has been in constant change, however, so was antibiotic resistance. Nowadays, it is thought that antimicrobial resistance lies as the cause of more than 700.000 yearly deaths globally, while hospital-acquired infection levels continue to rise in many parts of the world (Uddin et al., 2021). The last decades have witnessed a decreased interest of pharmaceutical companies regarding the development of novel antimicrobials, mostly due to the cost of production and poor return of investment in comparison with other drugs treating chronic disorders, for instance. Thus, the focus shifted toward the development of antimicrobial combinations, in addition to ongoing progress in the field of monoclonal antibodies and immunotherapy (Hutchings et al., 2019). Products such as cefiderocol (FDC), ceftolozane/tazobactam (C/T), ceftazidime/avibactam (CZA), imipenem/relebactam (IMR) aztreonam/avibactam (ATM-AVI) or sulbactam/durlobactam (SUL-DUR) have brought hope to both clinicians and the patients’ community by their promising efficacy rates in the case of difficult-to-treat infections. However, with most of these infections being caused by multidrug-resistant (MDR) or extended-drug-resistant (XDR) bacteria, these microorganisms have evolved in terms of resistance mechanisms even against these antimicrobial agents (Wang et al., 2020; Mushtaq et al., 2021; Teo et al., 2021; Gaibani et al., 2022; Karakonstantis et al., 2022). This review aims to briefly characterize these substances, highlighting the current patterns and mechanisms of resistance encountered *in vitro*.

## 2 Methods

**Figure 1:**
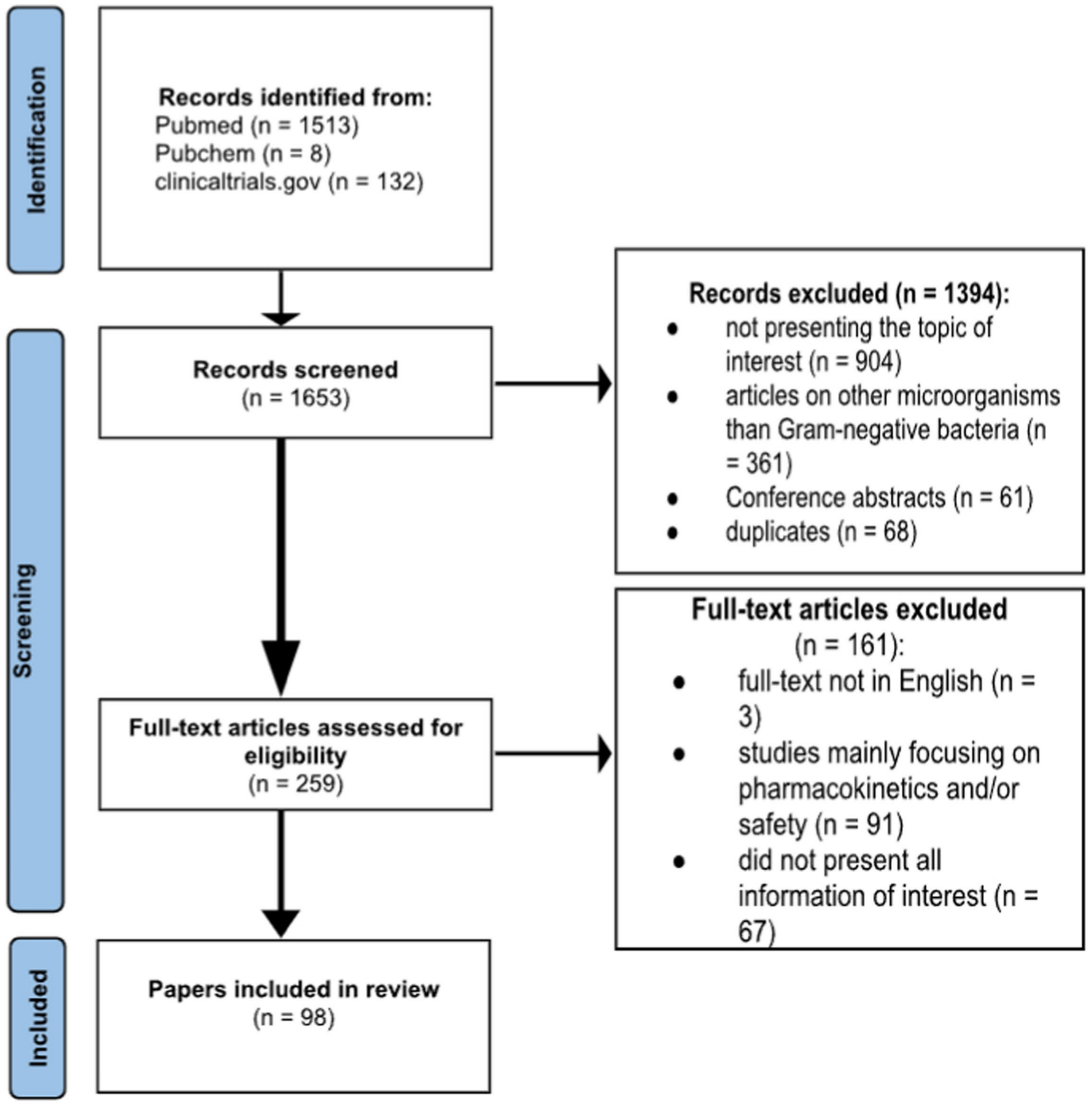
Flowchart of data collection, adapted from the PRISMA 2020 flow diagram.

The literature review was conducted independently by the authors in November–December 2023 based on the PRISMA 2020 guideline (Page et al., 2021). Articles from all years were sourced on PubMed. Systematic reviews, meta-analyses and clinical trials studying the desired 6 antimicrobials were included. General data about these antimicrobials, as well as resistance profiles of different Gram-negative pathogen strains against these agents have been studied. In addition, a search on PubChem has been conducted in order to obtain the chemical structures of the studied antibiotics. Moreover, a comprehensive assessment of clinical trials involving these antibiotics has been conducted using clinicaltrials.gov. Exclusion criteria were studies focusing only on pharmacokinetics and/or safety, articles presenting the antimicrobials in correlation with other bacterial pathogens. Keywords used in the search were ‘antimicrobial resistance,’ ‘ceftazidime/avibactam,’ ‘cefiderocol,’ ‘ceftolozane/tazobactam,’ ‘imipenem/relebactam,’ ‘aztreonam/avibactam,’ ‘sulbactam/durlobactam.’ The search formula used was “(antimicrobial resistance) AND [(ceftazidime/avibactam) OR (cefiderocol) OR (ceftolozane/tazobactam) OR (imipenem/relebactam) OR (aztreonam/avibactam) OR (sulbactam/durlobactam)].” Additionally, 10 articles have been selected for presenting further research directions, however the exclusion criteria were maintained. Search results were analyzed by all authors. PRISMA guidelines were followed, and bias risk was not assessed. In total, 1,653 records have been found, out of which 96 have been included as the references of this paper, following the screening criteria. This review has also been included in the International prospective register of systematic reviews (PROSPERO), with the registration ID CRD42024505832. The full flow of the scientific data collection can be consulted in Figure 1.

## 3 Results

### 3.1 General aspects

#### 3.1.1 Antimicrobial class and mechanism of action

##### 3.1.1.1 Cefiderocol

**Figure 2:**
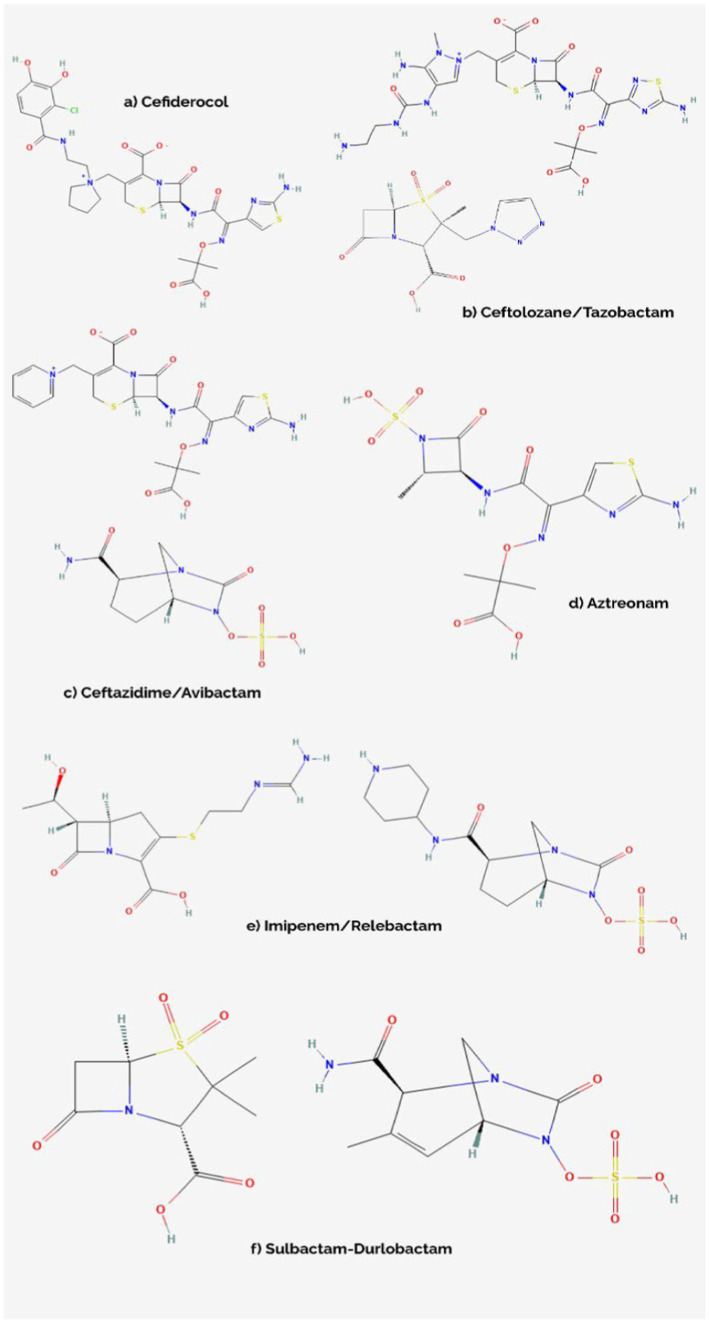
Chemical structures of cefiderocol **(A)**, ceftolozane/tazobactam **(B)**, ceftazidime/avibactam **(C)**, aztreonam **(D)**, imipenem/relebactam **(E)**, and sulbactam/durlobactam **(F)** (Avycaz, 2024; Aztreonam, 2024; Cefiderocol, 2024; Ceftolozane-Tazobactam, 2024; Durlobactam, 2024; Imipenem, 2024; Relebactam, 2024; Sulbactam, 2024).

FDC stands as an improved siderophore cephalosporin, sharing structural assets with both ceftazidime (shown in Figure 2), with which it shares the same C-7 side chain, and cefepime, from which it borrows the pyrrolidinium group on the C-3 chain, which translates into an improved stability level against *β*-lactamases such as KPC (*Klebsiella pneumoniae* carbapenemase), NDM (New Delhi metallo-*β*-lactamase), OXA (oxacillin carbapenemase) or VIM (Verona integron-encoded metallo-*β*-lactamase) (Table 1). In the case of the C-7 side chain, FDC showcases groups responsible for providing a certain level of stability against bacterial *β*-lactamases (the oxime and dimethyl groups), as well as an acidic component conferring improved permeability of outer membranes. The innovative structural feature is a chlorocatechol nucleus on the C-3 side chain, which gives cefiderocol the ability to chelate iron. Thus, FDC uses specific iron active transporters in microorganisms to pass through the outer membrane, in a mechanism known as a ‘Trojan horse’ strategy (Sato and Yamawaki, 2019; Karakonstantis et al., 2022). Moreover, besides being stable against the action of *β*-lactamases, FDC shows high affinity for penicillin binding proteins (PBPs), especially PBP3 but also *Klebsiella pneumoniae* PBP2 and *Pseudomonas aeruginosa* PBP1a (Ito et al., 2018). Thus, FDC manages to interfere with cell wall synthesis, resulting in cellular apoptosis (Sato and Yamawaki, 2019).

**Table 1:** Action comparison of FDC (cefiderocol), C/T (ceftolozane/tazobactam), CZA (ceftazidime/avibactam), IMR (imipenem/relebactam), ATM-AVI (aztreonam/avibactam), SUL- DUR (sulbactam/durlobactam) against selected *β*-lactamases.

Antimicrobial	Stable against			
	Amber Class A: KPC	Amber Class B: IMP, NDM, VIM	Amber Class C: AmpC	Amber Class D: OXA-48
FDC	Yes	Yes	Yes	Yes
C/T	No	No	Yes	Yes
CZA	Yes	No	Yes	Yes
IMR	Yes	No	Yes	Limited action
ATM-AVI	Yes	Yes	Yes	Yes
SUL-DUR	Yes	No	Yes	Yes

Adapted after (Van Duin and Bonomo, 2016). KPC, *Klebsiella pneumoniae* carbapenemase; VIM, Verona integron-encoded metallo-β-lactamase; NDM, New Delhi metallo-β-lactamase; IMP, imipenemase; OXA, oxacillinase; AmpC, cephalosporinase class C.

##### 3.1.1.2 Ceftolozane/tazobactam

C/T is a molecular association between a cephalosporin, ceftolozane, and a β-lactamase inhibitor, tazobactam. Ceftolozane is structurally similar to ceftazidime (shown in Figure 2) however showcasing increased stability against AmpC (cephalosporinase class C) β-lactamases – mediated hydrolysis, benefiting from a heavier side chain, which is vital when encountering *Pseudomonas aeruginosa* resistance mechanisms. Tazobactam, on the other hand, is a β-lactamase inhibitor with a β-lactam structural core, making it inefficient against some carbapenemases encountered in novel MDR/XDR bacteria, such as NDM-1, KPC-2, KPC-3 or OXA-48 (Table 1; Van Duin and Bonomo, 2016; Teo et al., 2021).

##### 3.1.1.3 Ceftazidime/avibactam

CZA is constituted of a third-generation cephalosporin, ceftazidime, a molecule sharing chemical characteristics with ceftolozane (shown in Figure 2), and avibactam, a β-lactamase inhibitor which does not belong to the β-lactam class, being a diazabicyclooctane substance. In addition to the properties of ceftazidime, which are similar to the other novel-generation cephalosporins, avibactam creates a reversible covalent bond with the serine residues belonging to the β-lactamase active center, which provides it with extended stability against β-lactamases, even against KPC-2, KPC-3 or OXA-48. However, it is not effective against metallo-β-lactamase (IMP, VIM, NDM) producing pathogens, due to the structural characteristics of their active sites, which do not contain serine residues (Table 1; Van Duin and Bonomo, 2016; Mosley et al., 2016; Gaibani et al., 2022).

##### 3.1.1.4 Imipenem/relebactam

IMR combines imipenem, a carbapenem which interferes with the process of cellular wall synthesis by inactivating PBPs contained by the membrane of the bacterial cell, with relebactam, another diazabicyclooctane similar to avibactam (shown in Figure 2), which limits the efflux of the complex via a side chain that presents positive charging. While shown to be effective against the likes of KPC or AmpC, relebactam has been demonstrated to be inactivated by class B metallo-β-lactamases (IMP, NDM, VIM), also showing limited action against OXA-48-like producing pathogens (Table 1; Heo, 2021; Gaibani et al., 2022; O’Donnell and Lodise, 2022).

##### 3.1.1.5 Aztreonam/avibactam

ATM-AVI is a molecular association currently in development, which combines aztreonam, a β-lactam agent stable against metallo-β-lactamase mediated hydrolysis, but otherwise susceptible to being degraded by ESBLs, KPCs or AmpCs, and avibactam, a diazabicyclooctane, which, by being effective against serine-containing β-lactamases, is considered to bring the final antimicrobial agent – ATM-AVI to a maximum level of effectiveness against pathogens producing most types of β-lactamases (Table 1; Mushtaq et al., 2021; Sader et al., 2023).

##### 3.1.1.6 Sulbactam/durlobactam

SUL-DUR, which has recently been approved by the FDA after successful results of phase III clinical trials, brings together two β-lactamase inhibitors – Sulbactam, one of the older antimicrobial agents, a penicillanic acid with limited anti-β-lactamase activity and Durlobactam, a synthetic diazabicyclooctane inhibiting class A, C and D β-lactamases. In terms of target sites, SUL targets PBP1a, PBP1b and PBP3, while PBP2 stands as DUR’s target. Together, they stand as a molecular association with efficient inhibitory activity against class A, C and D β-lactamase-producing *Acinetobacter baumannii* strains (Table 1; Keam, 2023; Karruli et al., 2023).

#### 3.1.2 Approval timeline and indications

Since the 1940s, development of antimicrobials helped medicine evolve beyond the treatment of infectious diseases, also enabling now common practices, such as invasive maneuvers, improve their safety and efficacy. However, bacteria fought back bringing us to the present public health concern that antimicrobial resistance represents, which combines with decreasing interest for novel antibiotic development on the pharmaceutical companies’ side, mainly due to increased research costs and rapid emergence of resistance, leading to low economic turnover. In 2017, a priority list of pathogens for which new antimicrobials’ research and development is needed was released by the WHO, placing carbapenem-resistant Enterobacteriaceae as third in the ranking for critical pathogens (World Health Organization, 2017). In accordance with these reports, the second half of the last decade has brought fruitful updates on the approval of novel antimicrobial agents used for the treatment of infections due to MDR/XDR Gram-negative bacteria, especially complicated urinary tract infections (cUTIs), complicated intra-abdominal infections (cIAIs), hospital-acquired pneumonia (HAP) and, respectively, ventilator-associated pneumonia (VAP) (Naseer et al., 2021; Yusuf et al., 2021). Moreover, CZA has also been approved for usage in the case of pediatric patients (older than 3 months) suffering from cUTIs and cIAIs, used in co-therapy with metronidazole (Bassetti et al., 2020). In addition, 2023 witnessed the FDA approval of SUL-DUR for adult patients suffering from *Acinetobacter baumannii* HAP/VAP (Keam, 2023). Nonetheless, ATM-AVI currently stands as a very promising candidate for FDA approval, with recent phase-3 clinical trials showcasing effectiveness and good tolerance levels when tested for the treatment of cIAI, HAP and VAP (Carmeli et al., 2023). The FDA approval timeline and indications for the 6 antimicrobials in our paper can be found in Table 2. However, these antimicrobials are not intended for firstline usage, due to the secondary risk of decreased efficacy caused by emergent resistance. Four of them (FDC, CZA, C/T and IMR) are classified in the ‘reserve’ group of antibiotics by the WHO, thus should be treated as ‘last resort’ options. ATM-AVI and SUL-DUR have not been included on this list due to their approval happening after the release of the WHO 2021 AWaRe classification (World Health Organization, 2021).

**Table 2:** FDA approval timeline and indications for cefiderocol, ceftolozane/tazobactam, ceftazidime/avibactam, imipenem/relebactam, aztreonam/avibactam, sulbactam/durlobactam including approval extensions for different pathologies and, in the case of ceftolozane/tazobactam, extension for pediatric use.

Antimicrobial agent	Time of approval	Indications	References
Cefiderocol	November 2019, September 2020	cUTI, HAP/VAP	Naseer et al. (2021) and Yusuf et al. (2021)
Ceftolozane/Tazobactam	December 2014, June 2019	cUTI, cIAI, HAP/VAP	Yusuf et al. (2021)
Ceftazidime/Avibactam	February 2015, February 2018, March 2019	cUTI (including pyelonephritis), cIAI (combined with metronidazole), HAP/VAP, including pediatric patients	Yusuf et al. (2021) and Bassetti et al. (2020)
Imipenem/Relebactam	July 2019, June 2020	cUTI, cIAI, HAP/VAP	Yusuf et al. (2021)
Aztreonam/Avibactam	Not yet approved	–	Sader et al. (2023)
Sulbactam-Durlobactam	May 2023	HAP/VAP caused by *Acinetobacter baumannii*- calcoaceticus complex organisms	Keam (2023)

FDA, Food and Drug Administration; cUTI, complicated Urinary Tract Infection; cIAI, complicated Intra-Abdominal Infection; HAP, hospital- acquired pneumonia; VAP, ventilator-associated pneumonia.

#### 3.1.3 Effectiveness

Multiple clinical trials have been conducted in patients suffering from cUTI, cIAI, HAP/VAP and even sepsis, focusing on the effectiveness of these antimicrobials in real-life scenarios, as well as comparing clinical and microbiological results with those of antibiotics that have been used in clinical practice beforehand. Comparative results present the fact that all these novel antimicrobials are non-inferior to previous therapies considered to be the best available. The full listing of these trials can be found in Table 3. In addition, we aim to discuss each antimicrobial’s particularities against MDR/XDR microorganisms below, as shown by previously conducted studies, both *in vitro* and *in vivo*.

**Table 3:** Results of clinical trials showing non-inferior or improved favorable clinical response rates of patients to treatment with FDC, C/T, CZA, IMR, ATM-AVI, and SUL-DUR as compared with other antimicrobials.

Antimicrobial	Trial (references)	Pathology	Comparator	Outcome rates (tested antimicrobial versus comparator)
FDC	Portsmouth et al. (2018)	cUTI	Imipenem/Cilastatin	Clinical cure: 73% versus 53%
FDC	Wunderink et al. (2021)	HAP, VAP, healthcare-associated pneumonia (HCAP)	Meropenem (high-dose, extended-infusion)	Clinical cure: 65% versus 67% Microbiological eradication: 41% versus 42%
FDC	Bassetti et al. (2021)	HAP, VAP, cUTI, sepsis, bloodstream infections (BSI)	Best available therapy	Clinical cure: HAP/VAP: 60% versus 63% cUTI: 77% versus 60% BSI/sepsis: 70% versus 50% Overall: 66% versus 58%
C/T	Kollef et al. (2019)	HAP, VAP	Meropenem	Clinical cure: 63.8% versus 64.7% Microbiological eradication: 73.1% versus 68%
C/T	Roilides et al. (2023)	cUTI, including pyelonephritis in pediatric patients	Meropenem	Clinical cure: 94.4% versus 100% Microbiological eradication: 93.0% versus 95.8%
C/T in combination with metronidazole	Sun et al. (2022)	cIAI	Meropenem	Clinical cure: 95.2% versus 93.1%
C/T	Chaftari et al. (2022)	Neutropenia and fever in patients with hematological malignancies	Standard-of-care	Clinical cure: 87% versus 72%
C/T in combination with metronidazole	Jackson et al. (2023)	cIAI in pediatric patients	Meropenem	Clinical cure: 80.0% versus 95.2%
C/T	Arakawa et al. (2019)	Uncomplicated pyelonephritis, cUTI	Non-comparative	Favorable clinical response rate: 96.6%
C/T in combination with metronidazole	Lucasti et al. (2014)	cIAI	Meropenem	Clinical cure: 83.6% versus 96%
C/T in combination with metronidazole	Mikamo et al. (2019)	cIAI	Non-comparative	Clinical cure: 92%
CZA	Bradley et al. (2019a)	cUTI in pediatric patients	Cefepime	Clinical response: 88.9% versus 82.6%
CZA in combination with metronidazole	Qin et al. (2017)	cIAI	Meropenem	Clinical cure: 93.8% versus 94.0%
CZA in combination with metronidazole	Lucasti et al. (2013)	cIAI	Meropenem	Clinical cure: 91.2% versus 93.4%
CZA in combination with metronidazole	Bradley et al. (2019b)	cIAI in pediatric patients	Meropenem	Clinical response: 91.8% versus 100%
CZA	Vazquez et al. (2012)	cUTI, including pyelonephritis	Imipenem/cilastatin	Favorable microbiological response: 70.4% versus 71.4%
CZA	Carmeli et al. (2016)	cUTI, cIAI	Best available therapy (in 97% of cases a carbapenem)	Clinical cure: 91.0% versus 91.0%
CZA	Torres et al. (2018)	HAP, VAP	Meropenem	Clinical cure: 68.8% versus 73%
CZA	Wagenlehner et al. (2016)	cUTI, including pyelonephritis	Doripenem	Combined symptomatic resolution + microbiological eradication: 71.2% versus 64.5%
CZA in combination with metronidazole	Mazuski et al. (2016)	cIAI	Meropenem	Clinical cure: 82.5% versus 84.9%
IMR	Kohno et al. (2021)	cIAI, cUTI	Non-comparative	Combined clinical cure: 94.02%
IMR	Sims et al. (2017)	cUTI	Imipenem	Microbiological response: 95.5% (when dosing 125 mg relebactam), 98.6% (when dosing 250 mg relebactam) versus 98.7%
IMR	Lucasti et al. (2016)	cIAI	Imipenem	Clinical response: 96.3% (125 mg relebactam), 98.8% (250 mg relebactam) versus 95.2%
IMR	Titov et al. (2021)	HAP, VAP	Piperacillin/Tazobactam	Clinical response: 61.0% versus 55.8%
IMR	Motsch et al. (2020)	HAP, VAP, cIAI, cUTI	Colistin + Imipenem	Favorable overall response: 71% versus 70%
ATM-AVI (in monotherapy and in association with metronidazole)	Carmeli et al. (2023)	cIAI, HAP, VAP	Meropenem +/− Colistin	Favorable microbiological response: 75.7% versus 73.9%
SUL-DUR	Kaye et al. (2023)	HAP, VAP, BSI caused by CRAB	Colistin	28-day all-cause mortality: 19% versus 32%
SUL-DUR in combination with imipenem	Sagan et al. (2020)	cUTI, including acute pyelonephritis	Placebo	Overall success: 76.6% versus 81%

FDC (cefiderocol), C/T (ceftolozane/tazobactam), CZA (ceftazidime/avibactam), IMR (imipenem/relebactam), ATM-AVI (aztreonam/avibactam), SUL-DUR (sulbactam/durlobactam), (cUTI) complicated Urinary Tract Infection, (cIAI) complicated Intra-Abdominal Infection, (HAP) hospital-acquired pneumonia, (VAP) ventilator-associated pneumonia.

##### 3.1.3.1 Cefiderocol

While clinical trials reported in Table 3 prove that FDC is non-inferior to its comparators, there have also been smaller studies focusing on its antimicrobial properties against MDR/XDR Gram-negative bacteria. For instance, a 2023 *in vitro* study conducted in the United Arab Emirates revealed that FDC has been highly efficient (97.9% efficacy) against MDR and XDR *Klebsiella pneumoniae* isolates, including carbapenemase producers and double carbapenemase-producers (NDM and OXA-48-like) (Daoud et al., 2023). Similar studies indicated that FDC has potent antimicrobial activity against the vast majority of isolates, including MDR and carbapenem-non-susceptible strains (Jacobs et al., 2018; Kazmierczak et al., 2019). The ARGONAUT-I study exhibited consistent susceptibility levels to FDC in multiple bacterial species, including non-fermenters: 97.0% in *Acinetobacter baumannii* complex strains, 100% amongst *Pseudomonas aeruginosa* and *Stenotrophomonas maltophilia* isolates (Jacobs et al., 2018).

##### 3.1.3.2 Ceftolozane/tazobactam

While C/T has received approval for several pathologies caused by Gram-negative pathogens, it is most commonly referred to as a viable treatment alternative for MDR/XDR *Pseudomonas aeruginosa* strains. Resistance patterns to C/T, as to most antimicrobials, vary from country to country, but recently published studies report effectiveness in more than 75% of cases (Venuti et al., 2023; Karlowsky et al., 2024; Mendes Pedro et al., 2024). However, a 2023 multicenter study focusing on various infections caused by MDR *Pseudomonas aeruginosa* (HAP, VAP, wound infections, UTI, IAI, catheter-related BSI) showcases that C/T does not provide significant differences regarding clinical outcome, when compared to CZA (Almangour et al., 2023). Additionally, an earlier multicentric study centering around gram-negative bacteria causing pneumonia concluded that, while C/T and CZA showed similar, encouraging susceptibility rates against *Pseudomonas aeruginosa* (96.0% for CZA and 95.9% for C/T), CZA was significantly more efficient against MDR Enterobacterales (99.2% susceptible to CZA, while only 53.8% susceptible to C/T) (Sader et al., 2020).

##### 3.1.3.3 Ceftazidime/avibactam

In addition to the 10 clinical trials concerning CZA included in Table 3, other studies reported significant efficacy of CZA against carbapenem-resistant and MDR Gram-negative pathogens. Wilson et al., focusing on CZA’s activity against *Pseudomonas aeruginosa* strains, concluded in a meta-analysis that this molecular association imposed a positive clinical outcome in 73% of infections with MDR or carbapenem-resistant *Pseudomonas aeruginosa* as etiologic agent (Wilson et al., 2021). Moreover, a study published in 2020 proved that over 90% of KPC-2 producing *Klebsiella pneumoniae* strains were susceptible to CZA, as well as this antimicrobial proving efficient against carbapenem-resistant *Pseudomonas aeruginosa* (Yang et al., 2020).

##### 3.1.3.4 Imipenem/relebactam

Data from published studies suggest that, while IMR proves as an efficient alternative when tackling MDR Enterobacterales (non-*Morganellaceae* Enterobacterales) such as *Escherichia coli*, *K. pneumoniae, Enterobacter* spp., *Citrobacter* spp. or *Serratia* spp., with a susceptibility rate of over 89% against these pathogens, it has shown low activity levels when encountering MBL-producing Enterobacterales, while also posing limited action against OXA-48-like producing Gram-negatives (Karlowsky et al., 2023). Moreover, IMR has proved inefficient against carbapenem-resistant *Acinetobacter baumannii* (CRAB), consistent with previous reports of CRAB resistance to imipenem (Mansour et al., 2021). Thus, IMR is now considered a viable treatment alternative when coming across several MDR Enterobacterales, unless encountering bacteria producing MBL or OXA-48 as an enzymatic resistance mechanism.

##### 3.1.3.5 Aztreonam/avibactam

The last years have seen multiple reports attempting to characterize the combination of aztreonam with avibactam in multiple clinical contexts. Nonetheless, reports show impressive susceptibility rates of Gram-negative bacteria to ATM-AVI (>97%), with this feature preserved in the MDR and XDR subgroups (Wise et al., 2023). However, with MDR/XDR/PDR microorganisms evolving in terms of resistance mechanisms, there is the need for more research in order to appropriately determine the activity of ATM-AVI against highly resistant strains. For instance, a 2023 study conducted in China concluded that ATM-AVI is indeed more efficient than CZA in MBL-producing XDR/PDR *Pseudomonas aeruginosa* isolates, but these microorganisms could potentially carry rare MBL encoding genes, thus it remains to be seen whether or not these resistance genes will become more commonly encountered in futurely described isolates (Kang et al., 2023). Other, smaller studies focused on more diverse Gram-negative microorganisms, such as *E. coli, K. pneumoniae, Enterobacter* spp., *Proteus mirabilis* or *Morganella morganii*. For instance, a 2021-published study assessing MBL-producing Gram-negative strains discovered that the addition of avibactam to aztreonam in previously not-susceptible strains results in significant *in-vitro* antimicrobial activity against 85% of strains (Bhatnagar et al., 2021).

##### 3.1.3.6 Sulbactam/durlobactam

SUL-DUR remains a highly efficient treatment alternative when treating infections caused by *Acinetobacter baumannii*. In a study characterizing globally collected strains from 2016 to 2017, SUL-DUR proved susceptible in over 97% of isolates, far more efficient compared to sulbactam alone (less than 50% susceptibility of isolates) (Karlowsky et al., 2022). When considering CRAB, SUL-DUR still shows promising results, with over 70% of CRAB isolates proving susceptible (Findlay et al., 2022).

### 3.2 Emergent resistance

Gram-negative pathogens exhibit resistance via 3 main mechanisms (enzyme-mediated antibiotic inactivation, structural changes of antimicrobial targets and cell permeability changes), closely linked to the active targets of antimicrobials and bacterial cell structure. Resistance can be intrinsic, when linked to chromosomal abnormalities in the bacterial genome, or acquired, via bacterial communication through transposable genetic elements, such as transferable plasmids (Breijyeh et al., 2020).

#### 3.2.1 Antibiotic inactivation through enzyme-mediated mechanisms

In accordance with the Ambler classification, there are 4 main categories of β-lactamases: A (which includes KPC and CTX-M, among others), B (which are known as the metallo-β-lactamases, such as IMP, VIM or NDM), C (AmpC and extended-spectrum variants), and D (which includes OXA). From a structural point of view, A, C and D groups contain serine residues at the enzyme’s active core, while class B showcases ions of zinc (Sawa et al., 2020). In this case, antimicrobial activity is diminished through the changes of structure in the enzymatic amino-acid chains, these mutant enzymes showcasing insertions or, most frequently, substitutions (Wang et al., 2020). Amino-acid substitutions have been comprehensively studied during the past years, consistent with increasing development in peptide engineering, in order to provide a better understanding of their role in antimicrobial interactions, especially concerning CZA and ATM-AVI. Notably, the amino-acid substitutions which can occur in the Ω-loop of β-lactamases, a structure playing a vital role in the catalytic activity of the enzyme, are linked to increased resistance to ceftazidime, via a mechanism known as ‘covalent trapping,’ leading to much faster hydrolysis of the substrate (Levitt et al., 2012). More specifically, studies focusing on the Asp179Asn substitution in the structure of KPC-2 confirmed that this mutation increases the MIC of CZA to these bacterial strains, as well as the fact that the addition of avibactam to ceftazidime does in fact increase efficacy against KPC-2 mediated resistance, but it remained insufficient to overcome the resistance conferred by the Asp179 mutations (Barnes et al., 2017). However, the same study showcased that ATM-AVI is efficient against these mutations-carrying variants. Another study revealed that there are no less than 65 structurally different KPC variants harboring resistance to CZA, with the majority of them (43/65) showcasing mutations in the aforementioned Ω-loop and 63% of them (41/65) also presenting insertions or deletions (Hobson et al., 2022). Moreover, resistance to carbapenems has been increasingly reported in AmpC presenting Enterobacterales (Breijyeh et al., 2020).

#### 3.2.2 Structural changes of the antimicrobial targets

Penicillin-binding proteins (PBPs) are protein structures that play a key role in bacterial wall synthesis, which makes them the target sites of FDC, C/T, CZA, IMR ATM-AVI and SUL-DUR. These structures, which can be divided into two categories taking into account their molecular weight - low-molecular weight PBPs and high-molecular weight PBPs, are inhibited by the antimicrobial substances they come in contact with, thus inhibiting the formation of peptidoglycan, a key component of the bacterial cell wall (Levitt et al., 2012). Structural changes at the PBP level have been reportedly linked to increased MIC levels, Alm et al. suggesting that a PBP3 four amino acid insertion is linked to decreased susceptibility to ATM-AVI of *Escherichia coli* (Alm et al., 2015). Moreover, studies show that the same mutation in *E. coli* isolates leads to elevated MIC levels of CZA (Wang et al., 2020). Additionally, mutations of PBP2, PBP3 and, morerarely, PBP1a and PBP1b are considered the most commonly encountered resistance pathways to SUL-DUR (Principe et al., 2022; Karruli et al., 2023). Ultimately, PBP-mediated resistance has been frequently highlighted in recent research, with most antimicrobials preferentially targeting one PBP with higher affinity, hence the antimicrobial combinations we have discussed having been constructed in order to inhibit more PBPs for utmost efficiency. The literature is scarce in describing the frequency of these structural changes’ occurrence, as it most certainly may be a multifactorial characteristic, however researchers consistently report that these modifications greatly impact the efficacy rates of antimicrobials (Sethuvel et al., 2023). Another potential mechanism of resistance is considered to be mutations in the structures of siderophore receptors1or iron transporters. These phenomena are of utmost importance when it comes to bacterial resistance against cefiderocol, due to its chemical properties.

Several studies have described the issue, highlighting multiple genes involved in iron transport pathways and the structure of the siderophore receptor (for instance: cirA, pirA, other TonB-dependent receptor genes, etc.) which can be potentially mutated or underexpressed, thus reducing intracellular uptake of cefiderocol and improving bacterial resistance (Domingues et al., 2023). While it is unsurprising that disturbances in iron metabolic pathway interact with cefiderocol’s antimicrobial activity, there have been studies reporting contradictory results, which imply the need for future biomolecular research highlighting the role of these structural changes in cefiderocol resistance (Karakonstantis et al., 2022).

#### 3.2.3 Cell permeability changes

Porins are outer membrane proteins that play a huge role in cellular permeability regulation, allowing hydrophilic substances to enter the cell via passive transport, necessary for cellular processes. Moreover, porins are also closely linked with the peptidoglycan in the bacterial wall structure, adding to their undeniable role in outer cover stability. Gram-negative bacteria present 5 porin types: OmpA, OmpC, OmpF, OmpW, OmpX. The loss of these porins and structural mutations have been shown to decrease antibiotic susceptibility in microorganisms presenting variants of these proteins, thus elevated MICs or even resistance rates have been observed in the case of most antimicrobials, including carbapenems, β-lactamases or third generation cephalosporins. Moreover, it has been shown that interactions between these porins also contribute to the development of resistance (Zhou et al., 2023). More specifically, taking *Klebsiella pneumoniae* as an example, outer membrane proteins OmpK35 and OmpK36 play a very important role in antimicrobial resistance, as there have been reported strong correlations between single or double deletions and increases in MIC levels against multiple antimicrobials (Tsai et al., 2011). Furthermore, it has been shown that homologous outer membrane proteins in different bacterial species present different characteristics. For example, Sugawara et al. discovered that OmpK35 and OmpK36 provide more efficient diffusion of β-lactams through the bacterial membrane than *E. coli* OmpF and OmpC, by creating channels with increased permeability and size (Sugawara et al., 2016).

Other bacterial components that contribute to permeability regulations are efflux pumps. While they can be linked to physiological processes, such as the elimination of cellular metabolites created on the course of respiration or the excretion of siderophores, bacterial-originating iron chelators, an essential adaptive mechanism to low-iron environments, efflux pumps also serve as a way of excreting antimicrobial substances, thus contributing to increased resistance. While they can either be chromosomally encoded or acquired via interbacterial communication, by transposons or plasmids, these structures are linked to complex intracellular regulation systems, grouping themselves into bacterial efflux systems, which can either excrete a specific antimicrobial, or more classes. For instance, efflux pumps belonging to the RND (resistance nodulation cell division) family excrete β-lactams, fluoroquinolones and linezolid, among others, while structures such as TetA are specific for tetracycline (Sharma et al., 2019; Nishino et al., 2021).

## 4 Discussion

### 4.1 Further directions

While these novel antimicrobial agents still present favorable effectiveness against a wide range of microorganisms, evolving resistance is a thing the healthcare community should be constantly aware of. Resistance mechanisms need to be further studied in order to be properly explored as potential targets for future therapeutic methods. Besides this, there is a constant need for novel strategies to be developed and, while antibiotic production finds itself at low levels, the focus is switching toward modern methods, such as antimicrobial combinations, immunotherapy, or molecule modeling. Considering the changes in the targets of antimicrobials, novel molecule development should be centered around updating stereospecific binding structures. Moreover, efflux pumps inhibitors are slowly shown as effective adjuvants or alternatives to antimicrobial agents in infections caused by resistant pathogens (Sharma et al., 2019). Nevertheless, the field of molecular science has been in constant evolution and novel approaches have shown promising results. One example of these is the development of dendrimers, nano-sized molecules previously researched for cancer treatment, which have recently been tested for antimicrobial activity via PBP affinity, showcasing promising results (Ahmed et al., 2016). Their silver salt structures have been increasingly studied in order to produce updated molecules, with improved MIC reduction capabilities, via elevated cationic characteristics through extra amino acid conjugation (Schito et al., 2021). More recently, molecular biology has further investigated the field of peptide-derived antibiotic development, which shall open a gateway to new-antimicrobial class discoveries in the future, by addressing the mechanisms of resistance that have been previously encountered, thus enabling the engineering of new means-of-action agents (Upert et al., 2021). After a study conducted both *in vitro* and on mouse models, Zosurabalpin now stands as a clinical candidate for the treatment of severe infections caused by CRAB. By successfully inhibiting the transport of lipopolysaccharide (LPS), more exactly the LptB2FGC complex, this drug provides alterations to the structural integrity of the bacterial cell. This discovery shines new light on how targeting LPS should be a viable strategy for treatment development against XDR/PDR (pandrug-resistant) microorganisms, however more extensive studies need to be performed (Pahil et al., 2024; Zampaloni et al., 2024).

Another interest-worthy field of advancements is represented by the usage of bacteriophages in order to efficiently treat these infections. These agents essentially act via a number of mechanisms, for instance pore creation and enzyme-mediated degradation of the bacterial peptidoglycan, resulting in bacterial cell lysis. While under development for human use, studies involving animal models have shown promising results, thus by adapting these treatments for human usage via improving pharmaceutical properties, we could see important progress in the field in the upcoming years (Zagaliotis et al., 2022).

Nonetheless, it is worth mentioning that there are several other antimicrobials undergoing testing for MDR Gram-negative organisms, such as cefepime/zidebactam or fosfomycin (Tirlangi et al., 2023; Meschiari et al., 2024). Also, while polymyxin analogs have not been previously taken into account when discussing the development of novel antimicrobials, there has been an increased interest in polymyxin engineering via amino-acid substitutions, thus it will be interesting to see how these substances will be able to perform *in vivo*, combined with the usage of machine learning and/or artificial intelligence applied to structuring of polymyxin analogs and other bioactive components (Li et al., 2021).

### 4.2 Limitations

Several limitations, although frequently encountered in other systematic review papers, need to be taken into consideration. Firstly, we have included only one scientific database into our research process. Even though we consider it to be the most appropriate for conducting a systematic review on our topic, some relevant information found in other databases might have been overlooked. Moreover, the quality of clinical trials included has not been assessed, as their selection was made based on relevance.

## 5 Conclusion

In a world which finds itself in continuous change, antimicrobial resistance still poses a consistent challenge for the healthcare & patients’ communities alike. The six antimicrobials that we have covered in this review are meant to be viable alternatives to the treatment schemes of MDR/XDR/PDR microorganism-borne infections. However, we believe that, in the light of recent emerging resistance of bacteria to these antibiotic agents, susceptibility rates need to be continuously monitored, in order to obtain an appropriate outlook on the course of treatment in each case. Additionally, more in-depth research is needed in order to fully understand the mechanisms of resistance against novel antimicrobials, as well as for finding updated means of tackling it. Nonetheless, antibiotic drug discovery should remain a priority for the healthcare industry in order for novel agents to become potential candidates for clinical usage, as well as alternative molecules arising from the studied means of resistance to be further developed.

## Data availability statement

The original contributions presented in the study are included in the article/supplementary material, further inquiries can be directed to the corresponding author.

## Author contributions

MD: Conceptualization, Data curation, Formal analysis, Investigation, Methodology, Resources, Validation, Visualization, Writing – original draft, Writing – review & editing. DT: Conceptualization, Data curation, Formal analysis, Investigation, Methodology, Supervision, Validation, Visualization, Writing – review & editing.
